# Impact of Dutch COVID-19 restrictive policy measures on physical activity behavior and identification of correlates of physical activity changes: a cohort study

**DOI:** 10.1186/s12889-022-12560-y

**Published:** 2022-01-21

**Authors:** Merle C. A. Schoofs, Esmée A. Bakker, Femke de Vries, Yvonne A. W. Hartman, Marcia Spoelder, Dick H. J. Thijssen, Thijs M. H. Eijsvogels, Laurien M. Buffart, Maria T. E. Hopman

**Affiliations:** 1 Radboud Institute for Health Sciences Department of Physiology, Radboud University Medical Center, Nijmegen, The Netherlands; 2 Research Institute for Sports and Exercise Sciences, Liverpool John Moores University, Liverpool, United Kingdom

**Keywords:** Coronavirus, Lockdown, Prevention, Physical inactivity, Healthy lifestyle, Netherlands

## Abstract

**Background:**

Identification of characteristics of individuals that are related to decreases in physical activity (PA) levels during lockdown is needed to develop targeted-interventions. This study aims to evaluate changes in domain-specific (i.e. leisure time, transportation, occupational, and household) and total PA due to the Dutch COVID-19 lockdown, which started on March 15 2020. Furthermore, we aim to identify demographic, health-related, and psychological correlates of these changes.

**Methods:**

Individuals who participated in the Nijmegen Exercise Study during 2017-2019 were invited to this study, which was conducted between April 16 and May 12 2020. Participant characteristics (i.e. age, sex, body mass index (BMI), marital status, education, household composition, and occupation status), living environment (i.e. housing type and degree of urbanization), psychological characteristics (i.e. resilience, outcome expectations, vitality, and mental health), and medical history were collected via an online questionnaire. Short Questionnaire to Assess Health-enhancing physical activity was used to assess PA behavior before and during lockdown. Wilcoxon signed-rank test was used to compare PA levels, in metabolic equivalent of task (MET)-minutes per week (min/wk), before and during lockdown. Multivariable linear regression analyses were performed to examine correlates of PA changes.

**Results:**

4033 participants (57% male; 59 ± 13 years) were included. PA decreased significantly during lockdown with mean ± SD changes of 393 ± 2735 MET-min/wk for total, 133 ± 785 MET-min/wk for transportation, 137 ± 1469 MET-min/wk for occupation, and 136 ± 1942 MET-min/wk for leisure time PA. Household PA did not change significantly. Unemployment, COVID-19-related occupational changes, higher BMI, and living in an apartment or semi-detached/terraced house were significantly related to larger decreases in total and domain-specific PA. Higher vitality was related to smaller decreases in total and domain-specific PA. Higher age was significantly associated with a larger decrease in leisure time PA. Lower education was associated with smaller decreases in transportation and occupational PA compared to higher education.

**Conclusion:**

PA levels significantly reduced during lockdown compared to before lockdown. Declines were observed during transportation and occupation, but were not compensated by an increase in leisure time PA. We identified subgroups that were more susceptible to reductions in domain-specific or total PA levels and should therefore be encouraged to increase their PA levels during lockdown.

**Supplementary Information:**

The online version contains supplementary material available at 10.1186/s12889-022-12560-y.

## Background

In December 2019, a new coronavirus causing severe acute respiratory syndrome emerged and spread rapidly throughout the world [1]. To reduce the spread of the coronavirus disease 2019 (COVID-19), many countries around the world implemented restrictive policy measures. On March 15 2020, the Dutch government issued a series of restrictions. Dutch residents were urged to stay at home as much as possible, keep a distance of 1.5 meters to others and work from home if possible. Sport clubs and fitness centers were closed, and public (sport) events were cancelled [2].

Chet et al. (2020) raised concerns that the COVID-19 protection measures might lead to reduced physical activity (PA) levels [3], which might increase the risk of premature death and non-communicable diseases, such as coronary heart disease, type 2 diabetes, and breast and colon cancer [4, 5]. A study performed in April 2020 supported this concern and indicated that half of the Dutch population reported to be less physically active since the COVID-19-lockdown [6]. Numerous international studies on the impact of the lockdown on PA have been conducted, which consistently demonstrated decreased in PA across several populations[7]. The magnitude of the decrease in PA in Dutch adults is however unknown.

Previous studies indicated that individuals with chronic conditions such as obesity, hypertension, and lung diseases, older individuals (≥55 years), and those without a garden are more likely to become physically inactive or perform less intense PA during lockdown [8, 9]. In addition to demographic and health-related correlates of PA behavior, also psychological variables such as resilience, vitality, and outcome expectations (i.e. beliefs or expectations about benefits of PA) may affect PA maintenance during lockdown as they have been positively associated with PA levels [10–12]. Four common PA domains in which PA occurs are leisure time, transportation, occupation, and household. These domains play a central role in understanding PA behavior, as decreases in one domain may be compensated by increases in other domains [13]. Associations between these correlates and changes in domain-specific PA are however lacking. Most studies focused on changes in total PA levels or exercise-related changes [8, 9, 14], whereas occupational and transportation PA are also important sources of total PA [15]. For example, in the Netherlands 29% of all trips to and from work were made by active transportation (i.e. cycling and walking) [16], suggesting that the increased number of hours working at home [17] might have drastic impact on individuals’ PA levels. Better understanding of correlates of changes in domain-specific PA behavior is necessary in order to identify individuals particularly at risk for decreases in PA levels. Identification of these individuals may aid the development of targeted-interventions aiming to maintain and increase PA levels of specific subgroups mostly affected by the lockdown [18, 19].

This study aims to evaluate the changes in total and domain-specific (i.e. leisure time, transportation, occupation, and household) moderate to vigorous intensity PA in Dutch adults as a consequence of the COVID-19 restrictive policy measures, and to identify the demographic, health-related and psychological correlates. We hypothesize that the restrictive policy measures lead to a reduction in total and domain-specific PA. Additionally, we hypothesize that individuals with health-related problems (such as higher body mass index (BMI) or a diagnosis with cancer, cardiometabolic or respiratory disease), older age, lower resilience, lower outcome expectations regarding the beneficial effects of PA, and those who started working from home have larger reductions in PA levels during the lockdown.

## Methods

### Study population and design

Participants of the Nijmegen Exercise Study [20] were invited via e-mail for an online questionnaire. The Nijmegen Exercise Study is a large prospective study examining the impact of PA on health, quality of life, and the development and progression of chronic diseases. This cohort consists of individuals participating in Dutch sport events (i.e. the Nijmegen Four Days Marches or the Seven Hills Run) and their family and friends. A subgroup of the study population (*n =* 9118) who completed the annual follow-up questionnaire in 2017-2019 was invited to this add-on study between April 16 and May 12 2020. Participants who were diagnosed with COVID-19 were excluded from the study (*n* = 35), as we were interested in the impact of the restrictive policy measures on PA, instead of the impact of a COVID-19 diagnosis on PA. All methods were performed in accordance with the relevant guidelines and regulations (Declaration of Helsinki). The Local Ethics Committee on Research Involving Human Subjects (CMO) of the region Arnhem and Nijmegen approved the study (#2011-193) and all subjects gave their informed consent before participation.

### Online questionnaire

Participants were asked about demographic characteristics, living environment, psychological characteristics, and PA behavior. General characteristics included age, sex, weight, marital status (i.e. 1) married or registered partnership, 2) unmarried/no partner), having children living at home (yes/no), education level, and occupation status. Education level was classified as 1) low: primary or prevocational secondary education, 2) intermediate: senior general, pre-university, or vocational education, and 3) high: higher or university education. Occupation status was classified as 1) unemployed, 2) employed without occupational changes since the lockdown, 3) employed and started (partially) working from home since the lockdown, and 4) employed and experienced other occupational changes since the start of the lockdown such as changes in working hours, work location (other than working from home), or work activities. Questions about the living environment were related to housing type (living in a detached house, semi-detached/terraced house, or apartment) and degree of urbanization (living in a rural, sub-urban, or urban area). Psychological characteristics included resilience, outcome expectations, vitality, and mental health. Resilience was measured using the Dutch version of the Resilience Evaluation Scale (RES), which varies from 0 to 36, with higher scores indicating greater psychological resilience [21]. Outcome expectations were assessed using the Multidimensional Outcome Expectations for Exercise Scale (MOEES) [22], which consisted of 15 statements divided over three domains (physical, social, and self-evaluative outcome expectations). The statements were assessed on a 5-point Likert-scale and were labelled as 1) completely disagree, 2) disagree, 3) neutral, 4) agree, and 5) completely agree. A total score was derived by summing the responses to each item and dividing by the total number of items, resulting in a score ranging between 0 and 100 with higher outcome expectation scores indicating a more positive outcome expectation regarding PA. Mental health and vitality were assessed with the respective subscales of the RAND-36 Health Survey (RAND-36) [23]. Scores vary from 0 to 100, with a higher score indicating better mental health and vitality.

The primary outcome was the difference between PA before and during the lockdown. PA before and during the lockdown (16 April – 12 May 2020) was assessed using the validated Short Questionnaire to Assess Health-enhancing physical activity (SQUASH) questionnaire [24]. Moderate to vigorous intensity PA volumes were expressed as metabolic equivalent of task minutes per week (MET-min/wk), and calculated for total PA and for subdomains leisure time, transportation, occupational, and household PA. Light intensity activities with a MET-value lower than 3 were excluded.

Data from previous data collections between 2017 and 2019 were used to assess medical history. We categorized participants as having comorbidities when they had a physician-confirmed diagnosis of cardiometabolic diseases (myocardial infarction, stroke, heart failure, hypertension or diabetes mellitus), cancer, or respiratory disease.

### Data analysis

All variables were visually inspected for normality and checked for skewness and by kurtosis. To explore the distribution of changes in PA levels, categories of change were based on the cut point 500 MET-min/wk, which is based on the 2018 US Physical Activity Guidelines [25]. Continuous variables were expressed as mean ± standard deviation when normally distributed, or as median [interquartile range: Q25-Q75] when not normally distributed. Categorical data were presented as numbers and proportions. Differences in PA levels between before and during the lockdown were compared using Wilcoxon signed-rank test since PA data were not normally distributed. A chi-squared test was performed to examine the impact of the lockdown on meeting the 2018 US Physical activity Guideline (i.e. 500 MET-min/wk of moderate to vigorous PA) [25]. We performed univariable and multivariable linear regression analyses to examine the relation between demographic, health-related and psychological characteristics and the change in total PA and domain-specific PA. In these models, PA levels during the lockdown were included as dependent variable and the PA levels before the lockdown as independent outcome variable in order to adjust for regression to the mean. To identify characteristics that were independently associated with PA level change, we applied backward selection. Variables selected as potential correlates were based on previous research on correlates of PA. All variables were entered in the model and subsequently removed one by one, starting with the variable with the highest p-value, until only significant (p < 0.05) correlates remained.

We used multiple imputation to impute missing values, since 7% of the individuals had missing data. We explored the pattern of missing data and assumed that data was missing at random. Participant characteristics did not significantly differ between participants with and without missing values. Multiple imputation by chained equations (MICE) using predictive mean matching was used. All available data were used to predict missing values in 10 imputed datasets with 100 iterations using the mice package [26].

P-values <0.05 were considered statistically significant. Analyses were carried out using R version 3.6.3.

## Results

### Study population

In total, 4068 (45%) participants filled in the online questionnaire of which 35 participants were excluded, because of a COVID-19 diagnosis resulting in a study population of 4033 participants. The majority of the participants were men (57%) and the mean ± SD (min-max) age of the participants was 59 ± 13 (20-90) years (Table 1). Most participants received higher education (57%) and 60% was employed. In total, 35% started (partially) working from home since the COVID-19-lockdown, 4% reported other occupational changes (e.g. changes in working hours, work location, and work activities), and 21% reported no occupational changes.

**Table 1:** Baseline characteristics of 4033 participants

**Characteristics**		*N* missing (%)
**Age, mean ± SD (min-max) years**	59 ± 13 (20-90)	0 (0.0%)
**Sex, n (%) female**	1721 (43)	0 (0.0%)
**Marital status, n (%) married or registered partnership**	3203 (80)	20 (0.5%)
**Having children living at home, n (%) yes**	655 (17)	64 (1.6%)
**Education, n (%)**		61 (1.5%)
*Low*	656 (17)	
*Intermediate*	1055 (27)	
*High*	2261 (57)	
**Occupation status, n (%)**		16 (0.4%)
*No changes*	885 (21)	
*Unemployed*	1593 (40)	
*Working from home*	1414 (35)	
*Other changes*	125 (4)	
**Housing type, n (%)**		8 (0.2%)
*Apartment*	652 (16)	
*Semi-detached / terraced house*	2381 (59)	
*Detached house*	992 (25)	
**Degree of urbanization, n (%)**		8 (0.2%)
*Urban*	1744 (43)	
*Sub-urban*	1375 (34)	
*Rural*	906 (23)	
**BMI, mean ± SD kg/m**^**2**^	24.2 ± 3.4	10 (0.2%)
**Outcome expectation score**^**1**^**, mean ± SD**	70.5 ± 12.4	13 (0.3%)
**Resilience score**^**2**^**, mean ± SD**	26.6 ± 5.4	11 (0.3%)
**RAND-Mental health score**^**1**^**, mean ± SD**	76.7 ± 13.9	14 (0.3%)
**RAND-Vitality score**^**1**^**, mean ± SD**	69.5 ± 15.8	15 (0.4%)
**Known comorbidities**^**3**^**, n (%) yes**	1834 (46)	52 (1.3%)

^1^Scores ranged from 0 to 100. ^2^ Score ranged from 0 to 36. ^3^ cardiometabolic diseases (myocardial infarction, stroke, heart failure, hypertension or diabetes mellitus), cancer, or respiratory disease

### Change in physical activity

**Fig. 1:**
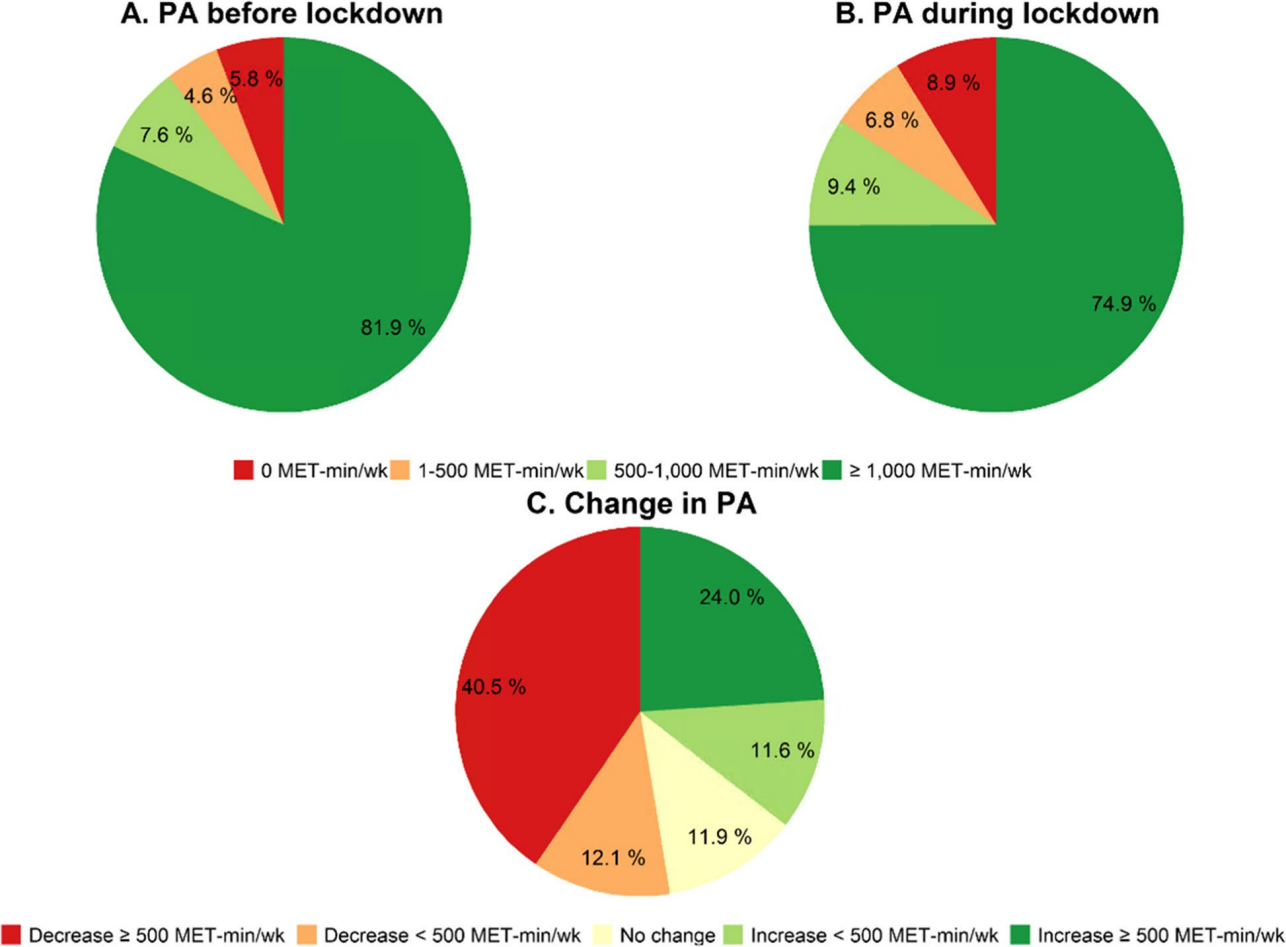
Pie charts of the distribution of PA before lockdown (**A**), PA during lockdown (**B**), and changes in PA levels (**C**)

The number of participants that met the threshold of 500 MET-min/wk of moderate to vigorous PA decreased significantly during the lockdown (p < 0.001). Before the lockdown, 3611 (90%) participants performed at least 500 MET-min/wk of moderate to vigorous PA (Fig. 1A), whereas this were 3403 (84%) participants during the lockdown (Fig. 1B). More than half of the population (53%) showed a decrease in PA levels during the lockdown. The largest group (41%) showed a decrease larger than 500 MET-min/wk. Almost a quarter (24%) of the population showed an increase larger than 500 MET-min/wk. No change in PA was reported by 12% of the population (Fig. 1C). Mean ± SD absolute change between total PA during the lockdown and before the lockdown was 393 ± 2735 MET-min/wk (*p* = 0.001). The mean absolute difference between domain-specific PA during the lockdown and before the lockdown was 136 ± 1942 for leisure time PA, 133 ± 785 for transportation PA, and 137 ± 1469 MET-min/wk for occupational PA (all p-values < 0.001). Household PA before the lockdown did not differ significantly from household PA during the lockdown (12 ± 550 MET-min/wk, *p* = 0.353) (Table 2).

**Table 2:** Physical activity levels in MET-min/week before and during the COVID-19 lockdown

	**PA before lockdown**	**PA during lockdown**	**PA before lockdown**	**PA during lockdown**	**Absolute difference**^**a**^	***P*-value**
	Median [IQR]	Median [IQR]	Mean ± SD	Mean ± SD	Mean ± SD	
**Total PA**	2928 [1440-4920]	2460 [996-4602]	3775 ± 3501	3382 ± 3437	393 ± 2735	<0.001
**Leisure time PA**	2082 [912-3516]	1826 [588-3486]	2568 ± 2365	2431 ± 2492	136 ± 1942	<0.001
**Transportation PA**	0 [0-340]	0 [0-0]	330 ± 739	198 ± 731	133 ± 785	<0.001
**Occupation PA**	0 [0-0]	0 [0-0]	583 ± 2118	446 ± 1894	137 ± 1469	<0.001
**Household PA**	0 [0-387]	0 [0-387]	295 ± 591	307 ± 620	12 ± 550	0.353

^a^Absolute difference between PA during lockdown – PA before lockdown. P-values represent Wilcoxon signed rank test

### Correlates of change in total physical activity

Results from the univariable linear regression analyses are presented in Additional file 1: Table S1. Multivariable linear regression analyses revealed that significantly larger decreases in total PA were found in participants who were unemployed (β -886, 95% CI -1093; -678 MET-min/wk), started working from home (β -910, 95% CI -1119; -702 MET-min/wk), or had occupational changes (β -1267, 95% CI 1724; 809 MET-min/wk) vs. no occupational changes, participants with a higher BMI (β -35, 95% CI -57; -13 MET-min/wk), and living in an apartment (β -691, 95% CI -935; -448 MET-min/wk), or semi-detached/terraced house (β -348, 95% CI -530; -165 MET-min/wk) vs. living detached. A smaller decrease was observed in those who are more vital (β 6, 95% CI 1; 11 MET-min/wk) (Table 3). Age, sex, marital status, having children living at home, education level, degree of urbanization, outcome expectation, resilience, mental health, and having comorbidities were not significantly associated with the change in total PA. Except for sex, results of the multiple imputation analyses were similar compared to the complete case analyses. Women vs. men (β -172, 95%C CI -332; -12 MET-min/wk) showed a significantly larger decrease in total PA in the complete-case analyses, but turned insignificant after multiple imputation.

**Table 3:** Multivariable linear regression models for the correlates of change in total and domain-specific PA

	**Total**	**Leisure time**	**Transportation**	**Occupational**
	β (95%CI)	β (95%CI)	β (95%CI)	β (95%CI)
**Age (years)**	-	-12 (-16; -7)	-	-
**Sex**				
*Male (ref)*			-	-
*Female*	-	-203 (-325; -82)		
**Education level**				
*High (ref)*	-	-		
*Intermediate*			69 (21; 117)	154 (60; 247)
*Low*			125 (66; 183)	165 (51; 278)
**Occupation status**				
*No changes (ref)*		-		
*Unemployed*	-886 (-1093; -678)		-290 (-344; -235)	-543 (-652; -434)
*Working from home*	-910 (-1119; -702)		-335 (-391; -279)	563 (-674; -451)
*Other changes*	-1267 (-1724; -809)		-353 (-473; -232)	-1158 (-1391; -924)
**Housing type**				
*Detached house (ref)*				-
*Semi-detached / terraced house*	-348 (-530; -165)	-241 (-376; -106)	-28 (-76; 20)	
*Apartment*	-691(-935; -448)	-522 (-704; -341)	-65 (-129; -1)	
**BMI (kg/m**^**2**^**)**	-35 (-57; -13)	-53 (-70; -36)	-	-
**RAND-Vitality score**	6 (1; 11)	6 (2; 9)	-	-
*Adjusted R*^*2*^	*0.49*	*0.48*	*0.22*	*0.56*

All models included correlates which were significantly related to total or domain-specific PA in the multivariable linear regression model. Adjustments were made for baseline PA levels. Ref = reference category

### Domain-specific correlates of change in physical activity

We found a larger decrease in leisure time PA in women vs. men (β -203, 95% CI -325; -82 MET-min/wk), participants living in an apartment (β -522, 95% CI -704; -341 MET-min/wk), or semi-detached/terraced house (β -241, 95% CI -376; -106 MET-min/wk) vs. living detached, participants with a higher BMI (β -53, 95% CI -70; -36 MET-min/wk), and older participants (β -12, 95% CI -16; -7 MET-min/wk). Smaller decreases were found in participants who were more vital (β 6, 95% CI 2; 9 MET-min/wk). Participants living in an apartment vs. living detached (β -65, 95% CI -129; -1 MET-min/wk), unemployed participants (β -290, 95% CI -344; -235), participants who started working from home (β -335, 95% CI -391; -279 MET-min/wk), or those who experienced occupational changes (β -353, 95% CI -473; -232 MET-min/wk) vs. employed participants without occupational changes showed a larger decrease in transportation PA. A smaller decline in transportation PA was observed in low vs. high-educated participants (β 125, 95% CI 66; 183 MET-min/wk), or intermediate vs. high-educated participants (β 69, 95% CI 21; 117 MET-min/wk). A larger decline in occupational PA was found in unemployed participants (β -543, 95% CI -652; -434 MET-min/wk), participants who started working from home (β -563, 95% CI -674; -451 MET-min/wk), or participants who experienced occupational changes (β -1158, 95% CI -1391; -924 MET-min/wk) vs. employed participants without occupational changes. A smaller decrease was observed in low vs. high-educated participants vs. low (β 165, 95% CI 51; 278 MET-min/wk), or intermediate vs. high-educated participants (β 154, 95% CI 60; 247 MET-min/wk) (Table 3). Multivariable linear regression results stratified by sex are presented in Additional file 1: Table S2.

## Discussion

This study revealed a negative change in PA levels during the Dutch lockdown. More than half of the study population showed a decrease in PA. More than 40% of the population reported a substantial (more than 500 MET-min/wk) decrease in PA and 24% reported an increase of more than 500 MET-min/wk. Declines were observed in the transportation and occupational domains. These domain-specific declines were not compensated by increasing leisure time PA. In fact, on average, leisure time PA levels also decreased during the lockdown. Second, our results suggest that the magnitude of the decline in PA levels differs among subgroups. In particular individuals with higher BMI, lower vitality, and who are unemployed are at risk for larger decreases in total PA. Also sex, age, education level, occupational changes, and housing type impacted domain-specific PA changes during the lockdown. This might indicate that interventions, to promote maintenance of individual’s PA levels during lockdown, should target specific subgroups which are mostly affected.

### PA changes during lockdown

Although the Dutch government issued a relatively mild lockdown compared to many other countries, and despite the recommendations to maintain physical activity during the lockdown [3], our results showed that the Dutch restrictions had a large impact on PA levels, which was similar to observations in other countries [14, 27, 28]. The mean decline of 393 MET-min/wk (i.e. almost 2 hours of walking at 4 km/h per week) may have detrimental public health effects. In the short-term, lower levels of PA may increase anxiety levels and reduce mental health [29, 30]. In the long-term, prolonged periods of lower levels of PA may increase the risk of chronic diseases [4, 5]. On the contrary, 36% of the population reported to be more physically active during lockdown, suggesting that reductions in PA levels occur in specific subgroups.

### Correlates of changes in PA during lockdown

Our finding that leisure-time PA levels were more affected in women than in men might be explained by gender differences in PA motivators and context preferences [31, 32]. For example, women more often prefer activities that are organized (i.e. supervised and at a fixed time) and performed indoors, and are motivated to maintain their PA levels when spending time with others. Indoor and joint PA activities were not allowed during the COVID-19-lockdown. Our finding is, however, in contrast to the results of a study performed in Sicily [14], where men experienced larger reductions in PA during the quarantine. This difference may be related to different policies, since Italian citizens were prohibited to venture further than 200 meters from their homes, thereby limiting PA outdoors [33].

The larger decreases in leisure time PA during the lockdown in older individuals and those with a higher BMI and the smaller decrease in leisure time PA in more vital individuals, might be explained by their increased risk of severe illness or death by COVID-19 infection [34]. The Dutch government advised individuals with high risk, such as elderly, those with comorbidities (e.g. cardiometabolic or respiratory disease), overweight or functional dependence to stay at home as much as possible [34]. Individuals’ stress and anxiety emerging from the risk of infection [35], may have hampered them to engage in leisure time PA, since stress is an important barrier towards exercise participation [36]. However, we found no significant association between comorbidity and the change in PA. The lack of associations may be explained by the relatively fit sample of individuals participating in sport events (i.e. the Nijmegen Four Days Marches or the Seven Hills Run), regardless of their comorbidity. Alternatively, we may have slightly underestimated the number of comorbidities as this information was collected 1 to 3 years ago. We were also unable to confirm our hypothesis that individuals with higher resilience, mental health, and outcome expectations showed a smaller decline in PA. This may be due to the relatively high resilience and mental health of the study sample.

Our finding that living in an apartment was related to a larger decline in leisure time PA compared to other housing types supports results from a previous study showing that individuals living in detached houses were more likely to be physically active [37]. These individuals have more opportunities to be physically active during gardening and housework in times of a lockdown [38]. Also, individuals living in an apartment showed a larger decline in transportation PA, which might relate to their physical environment. Detached houses are typically located in areas with a lower address density, whereas apartments are more frequent in regions with a higher address density. Active travel is generally higher in areas with a higher address density compared to lower density areas [39]. These lower density areas typically lack walking and cycling facilities, whereas these are highly present in urban areas [40]. Transportation PA levels of individuals living detached may already be lower before the lockdown and thereby less affected by the restrictive measures, in particular the working from home measure, in comparison to those living in an apartment with higher transportation PA levels before the lockdown.

We did not find a relation between level of education and change in total PA. It is however important to recognize that lower educated individuals showed smaller declines in transportation and occupational PA. Although we found no significant relation between education level and leisure time PA in the multivariable model, univariable analysis pointed out that lower educated individuals showed larger decreases in leisure time PA. This suggests that the direction of domain-specific changes in PA differ across different education levels and that these changes were compensated by increases in other domains that also differ between low- and high-educated individuals.

Our data also indicated that the increase in working from home and other occupational changes, such as changes in working hours, work location, and work activities, resulted in a decline in occupational and transportation PA. In our cohort, 45 percent of the employed population started to work (partially) from home during the lockdown. De Haas et al. (2020) reported that 27% of the Dutch workers that started to work from home expect to work from home more often in the future [17], suggesting that this might be an important target group to consider when developing intervention programs that promote PA in order to prevent the negative health consequences.

Previous studies suggested that also characteristics such as age, marital status, and having children living at home are related to the drop in total PA during lockdown. We found no evidence for such relation in our study. Differences between countries and populations may contribute to these distinct observations [41]. At least, in our data sample, these factors do not play a significant role.

### Strengths and limitations

Strengths of this study are the large sample size, the wide range of potential correlates and the ability to study domain-specific PA in addition to total PA. A limitation of our study is that our records of PA prior to the lockdown are collected retrospectively, which might have caused recall and over-reporting bias of PA [42]. However, we assume that the internal consistency, i.e. the consistency of participants’ responses to the questionnaire when repeated, of the participants led them to report similar bias for both before and during the lockdown. Therefore, we assume that our findings on the direction of changes in PA and thereby the identified correlates are still valid. A second limitation is the relatively low response rate (45%), which might have led to sampling bias, which possibly affects the generalizability of our findings. However, no significant differences in age and sex between responders and non-responders were found. Third, our study includes primarily physically active adults, which may limit the generalizability of the findings to the general Dutch population. Fourth, since our results are based on self-reported data, measurement error could have occurred. This might have resulted in a loss of precision and that associations were missed. Lastly, we did not adjust for weather or seasonality effects in our analyses. Pre-lockdown levels reflect individuals’ PA behavior during a winter month, whereas during-lockdown PA levels were assessed in spring. PA levels have been shown to be highest in spring and summer [43].We may therefore have underestimated the reduction in PA levels as a consequence of the lockdown.

## Conclusion

The current findings indicate a significant decline in PA behavior during the COVID-19 related lockdown compared to the period before the lockdown. More specifically, individuals who are unemployed, experienced occupational changes, live in an apartment or semi-detached/terraced house, and with a higher BMI showed larger reductions in total PA. Whereas individuals with a higher vitality showed smaller decreases in PA. We additionally identified domain-specific correlates of lockdown related changes in PA that could help to develop targeted-interventions aiming to maintain and increase PA levels or target those specific populations most at risk for reductions in PA levels during lockdown.

## Supplementary Information

**Additional file 1:** **Table S1.** Univariable linear regression models for the correlates of change in total and domain-specific PA. **Table S2.** Multivariable linear regression models for the correlates of change in total and domain-specific PA stratified by sex.

## Data Availability

The datasets used and/or analyzed during the current study are available from the corresponding author on reasonable request.

## Abbreviations

BMI: Body Mass Index
CI: Confidence Interval
COVID-19: Corona Virus Disease 2019
MET: Metabolic Equivalent of Task
MET-min/wk: MET-minutes per week
PA: Physical Activity
SD: Standard Deviation
