# Supplementary material for: Impact of Dutch COVID-19 restrictive policy measures on physical activity behavior and identification of correlates of physical activity changes: a cohort study
Source: BMC Public Health. 2022 Jan 21;22:147. doi: 10.1186/s12889-022-12560-y (PMC8777413; doi:10.1186/s12889-022-12560-y)
Supplement: Supplementary file 1 — Additional file 1: Table S1. Univariable linear regression models for the correlates of change in total and domain-specific PA. Table S2. Multivariable linear regression models for the correlates of change in total and domain-specific PA stratified by sex. [file 12889_2022_12560_MOESM1_ESM.docx]

**Additional file 1**

Table S1. Univariable linear regression models for the correlates of change in total and domain-specific PA.

|  | Total | Leisure time | Transportation | Occupational |
| --- | --- | --- | --- | --- |
|  | β (95%CI) | β (95%CI) | β (95%CI) | β (95%CI) |
| Age (years) | -4 (-10; 2) | **-8 (-12; -4)** | 1 (-1; 2) | 1 (-2; 4) |
| Sex  *Male (ref)*  *Female* | 7 (-148; 163) | -52 (-167; 62) | 36 (-5; 78) | -12 (-92; 68) |
| Marital status  *Not married or registered partnership (ref)*  *Married or registered partnership* | **277 (87; 469)** | **175 (35; 315)** | **54 (4; 105)** | 22 (-77; 120) |
| Having children living at home  *No (ref)*  *Yes* | 26 (-182; 234) | 128 (-24; 280) | -30 (-85; 26) | -70 (-177; 37) |
| Education level  *High (ref)*  *Intermediate*  *Low* | **206 (25; 387)**  112 (-103; 327) | -111 (-242; 21)  **-285 (-443; -127)** | **115 (68; 163)**  **165 (108; 222)** | **199 (106; 293)**  **199 (89; 309)** |
| Occupation status  *No changes (ref)*  *Unemployed*  *Working from home*  *Other changes* | **-862 (-1069; -654)**  **-918 (-1126; -709)**  **-1290 (-1750; -830)** | **-169 (-318; -19)**  13 (-139; 166)  188 (-151; 529) | **-290 (-345; -236)**  **-369 (-423; -315)**  **-351 (-471; -230)** | **-544 (-652; -436)**  **-610 (-719; -500)**  **-1149 (-1383; -915)** |
| Housing type  *Detached house (ref)*  *Semi-detached / terraced house*  *Apartment* | **-369 (-552; -185)**  **-748 (-994; -503** | **-216 (-351; -81)**  **-466 (-646; -285)** | -30 (-79; 19)  **-81 (-146; -16)** | -55 (-149; 40)  -77 (-204; 49) |
| Degree of urbanization  *Rural (ref)*  *Sub-urban*  *Urban* | **-237 (-446; -28)**  **-261 (-460; -61)** | -83 (-237; 69)  -56 (-202; 91) | -37 (-92; 18)  -31 (-84; 22) | -75 (-183; 32)  **-121 (-224; -18)** |
| BMI (kg/m^2^) | **-30 (-53; -8)** | **-52 (-67; -36)** | **8 (2; 14)** | **16 (5; 28)** |
| Outcome expectation score | 2 (-4; 8) | **7 (2; 11)** | **-2 (-4; -1)** | **-4 (-7; -1)** |
| Resilience score | 7 (-7; 21) | 8 (-3; 18) | -1 (-4; 3) | -1 (-8; 7) |
| RAND-Mental health score | 5 (-1; 10) | 3 (-1; 7) | 0 (0; 2) | 1 (-2; 4) |
| RAND-Vitality score | **7 (2; 12)** | **5 (1; 8)** | 1 (0; 2) | -1 (-3; 2) |
| Known comorbidities  *No (ref)*  *Yes* | -3 (-157; 152) | -57 (-170; 56) | 6 (-35; 47) | 6 (-73; 86) |

Bold values indicate significant correlations (p < 0.05). Ref = reference category

Table S2. Multivariable linear regression models for the correlates of change in total and domain-specific PA stratified by sex.

|  | Total | | Leisure time | | Transportation | | Occupation | |
| --- | --- | --- | --- | --- | --- | --- | --- | --- |
|  | Male | Female | Male | Female | Male | Female | Male | Female |
|  | β (95%CI) | β (95%CI) | β (95%CI) | β (95%CI) | β (95%CI) | β (95%CI) | β (95%CI) | β (95%CI) |
| Age (years) | - | - |  | -17 (-23; -10) | - | - | - | - |
| Education level  *High (ref)*  *Intermediate*  *Low* | - | - | - | - | 79 (18; 139)  105 (33; 177) | 64 (-15; 143)  173 (73; 272) | 129 (18; 241)  180 (46; 314) | - |
| Occupation status  *No changes (ref)*  *Unemployed*  *Working from home*  *Other changes* | -699 (-972; -427)  -744 (-1027; -462)  -793 (-1410; -176) | -1080 (-1408; -751)  -1106 (-1418; -795)  -1731 (-2415; -1047) | - | - | -247 (-317; -176)  -294 (-368; -220)  -302 (-461; -143) | -346 (-435; -256)  -387 (-474; -301)  -396 (-580; -212) | -377 (-510; -245)  - 332 (-472; - 192)  -551 (-845; -258) | -1080 (-1408; -750)  -1106 (-1418; -795)  -1731 (-2415; 1047) |
| Housing type  *Detached house (ref)*  *Semi-detached / terraced house*  *Apartment* | -260 (-492; -28)  -765 (-1085; -445) | -482 (-775; -190)  -648 (-1022; -273) | -186 (-364; -8)  -580 (-827; -333) | -305 (-510; -100)  -463 (-729; -197) | - | -61 (-147; 24)  -128 (-241; -14) | - | -482 (-775; -190)  -648 (-1022; -273) |
| Degree of urbanization  *Rural (ref)*  *Sub-urban*  *Urban* | - | - | - | - | - | 42 (-50; 134)  103 (3; 202) | - | - |
| BMI (kg/m^2^) | -51 (-81; -20) | - | -68 (-92; -45) | -38 (-62; -13) | - | - | - | - |
| Outcome expectations | - | - | 8 (1; 14) | - | - | - | -6 (-10; -2) | - |
| RAND-Vitality score | - | - | - | 7 (-23; -10) | - | - | - | - |

All models included correlates which were significantly related to total or domain-specific PA in the multivariable linear regression model. Adjustments were made for baseline PA levels. Ref = reference category
